# Delayed development of basic numerical skills in children with developmental dyscalculia

**DOI:** 10.3389/fpsyg.2023.1187785

**Published:** 2024-01-11

**Authors:** Sarah Lamb, Florian Krieger, Jörg-Tobias Kuhn

**Affiliations:** Department of Rehabilitation Sciences, TU Dortmund University, Dortmund, Germany

**Keywords:** developmental dyscalculia, basic numerical skills, domain-specific deficits, dot enumeration, magnitude comparison, number sets, number line

## Abstract

Research suggests that children with developmental dyscalculia (DD) have deficits in basic numerical skills. However, there is conflicting evidence on whether basic numerical skills in children with DD are qualitatively different from those in typically developing children (TD) or whether basic numerical skills development in children with DD is simply delayed. In addition, there are also competing hypotheses about deficits in basic numerical skills, assuming (1) a general deficit in representing numerosities (Approximate Number System, ANS), (2) specific deficits in an object-based attentional system (Object Tracking System, OTS), or (3) deficits in accessing numerosities from symbols (Access Deficit, AD). Hence, the purpose of this study was to investigate whether deficits in basic numerical skills in children with DD are more indicative of a developmental delay or a dyscalculia-specific qualitative deviation and whether these deficits result from (selective) impairment of core cognitive systems involved in numerical processing. To address this, we tested 480 children (68 DD and 412 TD) in the 2nd, 3rd, and 4th grades with different paradigms for basic numerical skills (subitizing, counting, magnitude comparison tasks, number sets, and number line estimation tasks). The results revealed that DD children’s impairments did not indicate qualitatively different basic numerical skills but instead pointed to a specific developmental delay, with the exception of dot enumeration. This result was corroborated when comparing mathematical profiles of DD children in 4th grade and TD children in 2nd grade, suggesting that DD children were developmentally delayed and not qualitatively different. In addition, specific deficits in core markers of numeracy in children with DD supported the ANS deficit rather than the AD and OTS deficit hypothesis.

## 1 Introduction

According to DSM-5 (American Psychiatric Association, 2013) and ICD-11 (World Health Organization, 2019), developmental dyscalculia (DD) is classified as a specific learning disorder characterized by several impairments in acquiring mathematical competency compared to typically developing children (TD) with a prevalence of 3–7% depending on diagnostic criteria (e.g., Moll et al., 2014). Common definitions agree that low mathematical achievement (i.e., more than one standard deviation below average) must occur despite adequate education and normal intelligence (i.e., not more than two standard deviations below average; e.g., Szardenings et al., 2018). While a cutoff at the 25th percentile in a mathematical achievement test identifies children with learning difficulties with a broad etiological basis, a stringent cutoff (e.g., ≤ 10th percentile) more likely comprises children whose underachievement is associated with neurobiological deficits, for example, children with DD (see Mazzocco et al., 2011).

The key causes of DD continue to be debated (e.g., Decarli et al., 2023). While some authors assume that domain-general deficits play a key role (e.g., Geary, 2004), others suppose that deficits in domain-specific skills, especially in basic numerical skills, lie at the core of DD (e.g., Butterworth, 2010). In the current study, we will focus on deficits in basic numerical skills as key causes of DD.

Although current research mostly shows that deficits in basic numerical skills characterize DD (Butterworth et al., 2011), there is still conflicting evidence on whether these deficits indicate qualitative differences in basic numerical skills between DD and TD or whether DD children’s skills are not qualitatively different but rather developmentally delayed. Most findings point to lower efficiency (e.g., Schwenk et al., 2017) and accuracy (e.g., Geary et al., 2008) in numerical processing, suggesting a developmentally delayed basic numerical profile in children with DD. Other findings show that the basic numerical skills of children with DD are persistently qualitatively different (e.g., Landerl, 2013), indicating that children with DD have a disproportionate impairment in basic numerical skills, resulting in abnormal numerical cognition (Landerl, 2013).

The situation is comparable concerning evidence at the neuronal level for children with DD. Studies show that children with DD display persistent structural and functional brain anomalies (Rotzer et al., 2008; Kaufmann et al., 2011; McCaskey et al., 2020). However, it remains unclear whether these abnormalities are due to developmental delay or specific markers of DD (McCaskey et al., 2020).

Based on the ability-level-match approach proposed by Bradley and Bryant (1978), the current study addresses whether DD children’s impairments in basic numerical skills are more likely to indicate a developmental delay or a dyscalculia-specific qualitative deviation. This approach suggests that children’s development is delayed if children with low abilities differ from their TD peers but are similar to younger children with the same ability. Alternatively, if the performance of low-achieving children differs from their TD peers and younger children with the same ability, this indicates a qualitatively different development pattern. The idea of an ability-level-match design, well known from reading-related research (e.g., Cain et al., 2000), has been applied in other fields as well. For example, Poloczek et al. (2012) investigated working memory in children with intellectual disabilities compared to matched TD children of the same mental age. In a study focusing on mathematical strategy development, Torbeyns et al. (2004) found differences in strategy use among 2nd grade children with strong and low mathematical abilities but not between 2nd and 3rd grade children matched on mathematical ability. This suggests that the mathematical strategies of children with low mathematical abilities reflect an immature level of numerical ability characterized by a delay (see Li et al., 2020).

In DD research, this approach has been used less frequently. Skagerlund and Träff (2014) examined the basic numerical skills of DD children in 4th grade, compared to an age-matched control group of TD children in 4th grade and a math ability-matched control group of TD children in 2nd grade. Based on reaction times (RTs), no significant differences were found between the DD and TD groups. Although descriptive, DD children showed lower RTs than TD children in 2nd grade but longer RTs than TD children in 4th grade, suggesting that the abnormalities are due to developmental delay. In the most recent studies, qualitative deviations were inferred based on statistical interactions or varying slopes. However, it is important to take age into account to ensure that qualitatively different deviations are not moderated by age-related differences. There are substantial changes in children’s mathematical development in the first years of schooling (e.g., Landerl, 2013). Especially in primary school, differences in mathematical skills between TD and DD children can, therefore, be influenced by children’s numerical age development. Thus, the question of whether DD is related to a qualitative difference or a delay can ultimately only be answered if we take children’s numerical age development (grade level) into account. Statistical evidence of dyscalculia-specific *qualitative* impairments refers to an (over-additive) statistical interaction effect between group (TD/DD) and basic numerical skills, as well as the absence of interactions moderated by grade level. In the case of *delayed* (additive) impairment, the performance of children with DD is generally slower or less accurate than that of children without DD, but there are no qualitative differences between the groups. This is reflected in the lack of statistical interaction effects.

Strongly intertwined with the characterization of basic numerical deficits (qualitative difference in DD or TD or developmental delay of DD children) is the question about the causes of basic numerical deficits. In fact, there are conflicting hypotheses and heterogeneous results pertaining to the causes of basic numerical deficits. These hypotheses consider deficits in core systems for numerical processing and derive predictions on how deficits in one of these systems affect basic numerical skills in general.

Due to the heterogeneous evidence on characterization and causes of impaired basic numerical skills, the goals of this study were to comprehensively investigate (1) whether DD children’s impairments in basic numerical skills are more likely to indicate a developmental delay or a dyscalculia-specific qualitative deviation and (2) whether these deficits derive from a (selective) impairment in cognitive core systems of numerical processing. Answering these research questions is essential to design and improve interventions for children with DD. We selected groups of children who matched in chronological school age (2nd, 3rd, and 4th grades) but who differed in mathematical ability to compare their performance in basic numerical skills in a cross-sectional design. Inspired by the ability-level-match design approach and similar to Skagerlund and Träff (2014), we compared the basic numerical profiles of 4th grade DD children with the performance of 2nd grade TD children to examine whether DD is a developmental delay or results in a qualitatively different numerical processing profile. In the following, we will describe core systems of numerical processing.

### 1.1 Core systems of numerical processing

**Figure 1:**
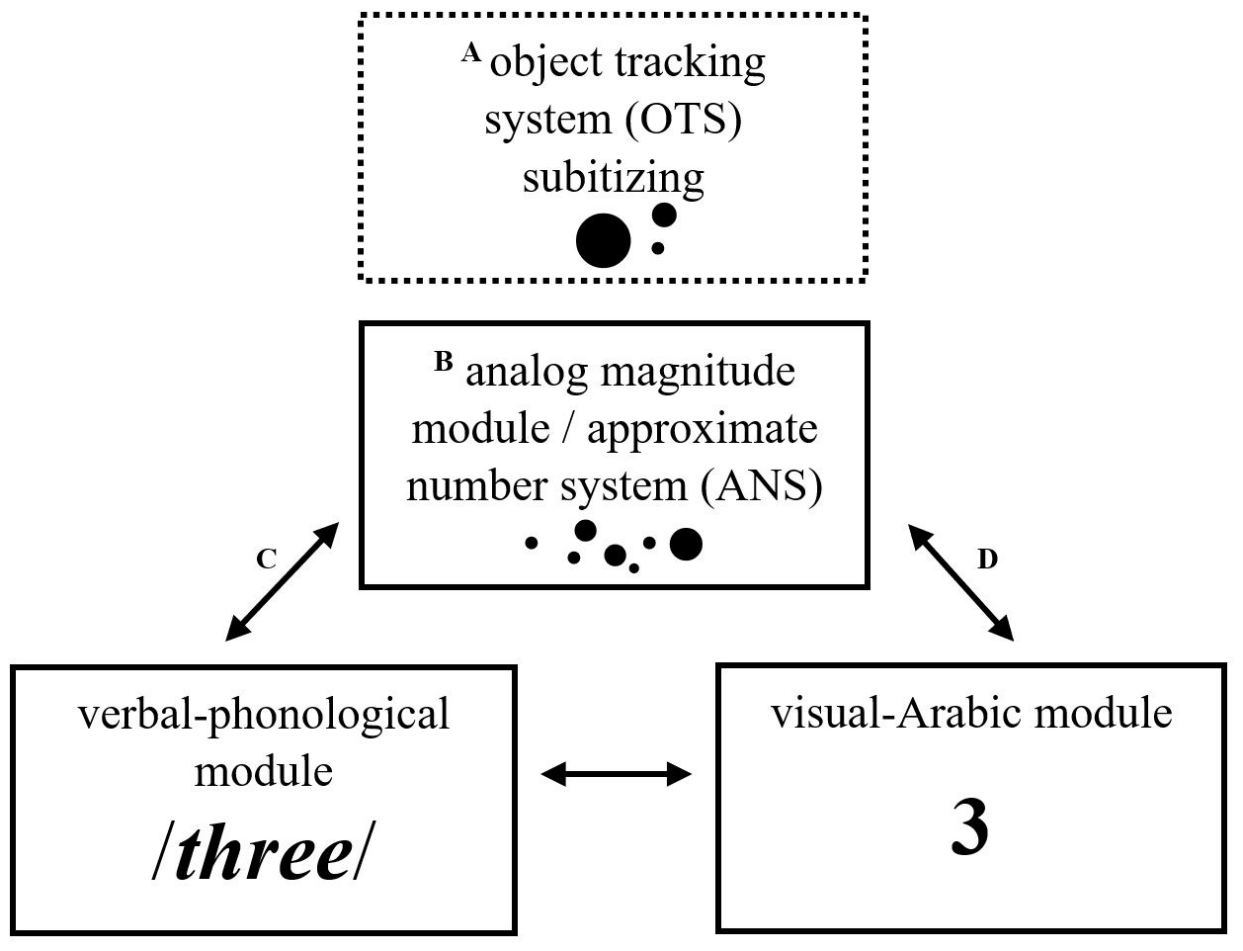
Core systems of numerical processing. Object tracking system (OTS) and approximate number system (ANS) are seen as innate, independent core systems of numerical processing (Feigenson et al., 2004). ANS/analog magnitude, verbal-phonological, and visual-Arabic modules are regarded as neurocognitive numerical processing components of the Triple Code Model by Dehaene (1992). The letters pertain to the hypotheses about the causes of developmental dyscalculia. **(A)** OTS deficit = specific deficit in subitizing; **(B)** ANS deficit = general deficit in representing numerosities; **(C,D)** Access deficit (AD) = deficits in accessing numerosities from symbols (based on Landerl et al., 2021).

Feigenson et al. (2004) distinguish two independent core systems of numerical processing. One system is used for *approximate* recognition and estimation of numerosities (approximate number system, ANS). In contrast, the other core system (object tracking system, OTS) is utilized for *accurate* discrimination and recognition of three to four objects without counting them (*subitizing*). These innate core systems are the foundation for developing the symbolic number system (Feigenson et al., 2004). According to the Triple Code Model (TCM) by Dehaene (1992), building on the ANS that processes large (> 4) representations of non-symbolic numerosities (e.g., •••), children develop two additional modules of numerical processing during preschool and school (von Aster and Shalev, 2007): the verbal-phonological module is responsible for processing written and spoken number words (e.g., /three/), whereas the visual-Arabic module processes written Arabic numerals (e.g., 3). Several theories propose that the cause of DD could be a deficit in one of the core systems of numerical processing (see Figure 1).

According to the *OTS deficit hypothesis*, some authors see the cause for DD in a defective OTS (e.g., Butterworth, 2010; see Figure 1A), and according to the *ANS deficit hypothesis,* some authors see the cause in a defective ANS but non-impaired OTS (e.g., Piazza et al., 2010; see Figure 1B). In addition, the *access deficit hypothesis* (AD) (e.g., Rousselle and Noël, 2007; see Figures 1C,D) claims that the ANS is not impaired *per se*, but *accessing* the visual Arabic module is impaired, which indicates an access deficit. Thus, DD children should be (disproportionately) impaired in symbolic numerical processing compared to non-symbolic numerical processing (see Noël and Rousselle, 2011; Schwenk et al., 2017).

Because the development of numerical processing is based on the core systems (e.g., Wilson and Dehaene, 2007), all three hypotheses predict problems in processing representations from symbols. However, the findings pertaining to non-symbolic numerical processing are less clear. While some results support the ANS hypothesis (e.g., Decarli et al., 2020), others are more in line with the AD hypothesis (e.g., Schwenk et al., 2017). In addition to a lack of consistent criteria and methods to critically assess empirical results with respect to these hypotheses (see Olsson et al., 2016), age could explain the conflicting results. According to Rousselle and Noël (2007), deficits in processing non-symbolic numerosities could also be interpreted as a consequence of (poor) mathematical development. A review by de Smedt et al. (2013) revealed that 9-year-old or older children with DD showed more inefficient non-symbolic numerical processing than TD children; younger DD children, however, did not. This result points to different assumptions about the causes of DD depending on a child’s age. Nevertheless, especially in primary school, age could be an influencing factor, as early math learning represents a sensitive developmental phase for mathematical competencies (see Raddatz et al., 2017). Children are increasingly detached from visual materials and the number space expands, and they are taught the basics of arithmetic (see Landerl, 2013). This is reflected in developmental leaps in basic numerical skills in the first years of schooling (e.g., Moore and Ashcraft, 2015) and in increasing efficiency and accuracy in performing basic numerical tasks from grade to grade (see Landerl and Kölle, 2009; Landerl, 2013; Moore and Ashcraft, 2015). In the 2nd grade, children still stand at the very beginning of their mathematical education. In the 4th grade, in contrast, they have already reached an adult-like level in some basic numerical tasks (Moore and Ashcraft, 2015). Thus, there are particularly large developmental leaps from 2nd grade to 4th grade.

Importantly, previous studies (e.g., Andersson and Östergren, 2012; Decarli et al., 2023) that contrasted hypotheses referring to the causes of DD often did not consider age-related differences in primary school children. In turn, many studies focusing on DD children’s numerical age development failed to systematically compare the hypotheses about the causes of DD (e.g., Landerl, 2013) or investigated only a small spectrum of basic numerical skills (e.g., Landerl and Kölle, 2009; Schleifer and Landerl, 2011). However, these studies did not explicitly investigate whether DD children’s basic numerical skills are qualitatively different or, instead, developmentally delayed. Other studies either only considered DD children in 4th grade (Skagerlund and Träff, 2014) or did not compare basic numerical profiles of 4th grade DD children and 2nd grade TD children (e.g., Landerl, 2013).

To summarize, the question of whether numerical processing deficits in children with DD stem from an ANS and/or OTS deficit or whether they can be explained by the AD hypothesis has not yet been conclusively answered. Furthermore, we cannot conclusively say whether DD children’s deficits are more indicative of a developmental delay or a qualitatively different numerical deficit profile. Addressing this gap, we presented different basic numerical tasks to children with and without DD, allowing a detailed view of numerical processing. The following subsection introduces the state of research on basic numerical deficits in DD along the different numerical processing task paradigms (see Landerl, 2019) investigated in the current study.

### 1.2 Subitizing vs. counting

To investigate whether DD children are impaired in numerical judgments of small countable objects, enumeration tasks are typically used. These tasks test the ability to count a limited number of objects (e.g., dots) as rapidly as possible (e.g., Landerl, 2019). About three to four dots can be processed in a preattentive way by tapping the OTS (subitizing), while more than three to four dots require serial counting (Trick and Pylyshyn, 1993) by tapping the verbal-phonological module. The latter is related to the ANS (von Aster and Shalev, 2007) in that lower efficiency in counting also suggests a defective ANS.

Several studies report subitizing and/or counting problems for DD children (Schleifer and Landerl, 2011; Andersson and Östergren, 2012; Landerl, 2013; Decarli et al., 2023). Some results (e.g., Landerl et al., 2004; Raddatz et al., 2017) suggest that DD children have a qualitatively different approach to counting, while other results (e.g., Schleifer and Landerl, 2011; Landerl, 2013) indicate a dyscalculia-specific impairment in subitizing, suggesting that DD children need to count even three or fewer dots serially (Kuhn et al., 2013). Generally, both subitizing and counting skills steadily increase across children’s development (Reeve et al., 2012; Moore and Ashcraft, 2015). However, while TD children become systematically more efficient at subitizing, there is evidence that this is not the case for DD children (Landerl, 2013). Previous study results showed that children with DD display more difficulties and larger slopes in the subitizing range than TD children (e.g., Schleifer and Landerl, 2011; Landerl, 2013), suggesting that the RTs of the DD children were not only slower but disproportionately slower, indicating qualitatively different numerical processing. Consistent throughout elementary school, this difference is an indication of a different development in DD and a dyscalculia-specific subitizing problem (Landerl, 2013). Although DD children were also substantially more inefficient in the counting range than TD children, the developmental trajectory did not appear to be qualitatively different (Schleifer and Landerl, 2011; Landerl, 2013).

### 1.3 Non-symbolic, symbolic, and mixed magnitude comparison

The most common method of generating information about the cognitive representation of numbers and the precision or efficiency of the ANS is to use magnitude comparison tasks (e.g., Halberda et al., 2008). This task type involves selecting the numerically larger of two (non-)symbolic numerosities (e.g., dots or numbers) as quickly as possible without counting (e.g., Moyer and Landauer, 1967; Halberda et al., 2008; Landerl, 2019). The estimation of numerosities is based on the ANS (Feigenson et al., 2004). The mental representation of numerosities in children with DD is thought to be less precise than in children with TD (e.g., Piazza et al., 2010), resulting in less correct differentiation of close numbers (also known as *distance effect*) by DD than by TD children. As a result, children with DD need longer RTs and/or show lower accuracy. In line with the ANS deficit, DD children should display impaired performance in non-symbolic magnitude comparison tasks (see Wilson and Dehaene, 2007). In contrast to the comparison of non-symbolic quantities, before comparing symbolic quantities (i.e., numbers), the visual-Arabic input must be converted to the analog quantity (Henik and Tzelgov, 1982). Thus, there is an interaction between the ANS and the visual-Arabic module, which, according to the AD hypothesis, is disturbed in DD. Some studies support the ANS deficit hypothesis (e.g., Feigenson et al., 2004; Piazza et al., 2010; Decarli et al., 2020), while other studies show that children with DD are only impaired in symbolic comparisons or at least have difficulty in non-symbolic comparisons to a much lesser extent (e.g., Olsson et al., 2016; Schwenk et al., 2017), thus supporting the AD hypothesis.

Age seems to be an important moderator of the results. Children with and without DD generally become more efficient with increasing age (Landerl, 2013). However, the development of symbolic numerical processing in DD children proceeds less systematically than in TD children (Landerl, 2013). In line with the ANS deficit hypothesis, Skagerlund and Träff (2014) revealed that the DD children showed significantly noisier ANS representations than TD children in the 4th and 2nd grades (Skagerlund and Träff, 2014). Piazza et al. (2010) observed that 10-year-old DD children’s number acuity was comparable to that of TD children 5 years younger, suggesting a delayed mathematical development. Several studies reported differences in non-symbolic comparisons only between older TD and DD children, but not in younger children with and without DD (for a review, see de Smedt et al., 2013), supporting the AD hypothesis by Rousselle and Noël (2007), which interprets that non-symbolic deficits occur as a consequence of (poor) mathematical development. In summary, age seems to influence DD and TD children’s performance in non-symbolic processing, but the evidence remains unclear (e.g., de Smedt et al., 2013; Skagerlund and Träff, 2014).

#### 1.3.1 Comparison distance effect

In addition, in the context of the magnitude comparison task, it has reliably been reported that the larger the distance between the two quantities (e.g., dots or numbers) being compared, the better the observed performance (e.g., Moyer and Landauer, 1967; Henik and Tzelgov, 1982). This finding is referred to as the comparison distance effect (Moyer and Landauer, 1967; Dehaene, 1992; Holloway and Ansari, 2008). In line with the most common interpretation, the distance effect is based on the assumption that cognitive magnitude representations are ordered along a mental number line/or mapped on the ANS (see also mental number line theory; Dehaene, 2011). It follows that the distance effect is also an indicator of the ANS acuity (e.g., Peters et al., 2008). Due to the ANS deficit, DD children should have a mental number line that is less mature, making it more challenging to represent and distinguish (non)-symbolic magnitudes (e.g., Mussolin et al., 2010; Piazza et al., 2010; Andersson and Östergren, 2012). Consequently, DD children should also display a greater distance effect than TD children. However, research results concerning the distance effect are not consistent. In line with the ANS deficit, a larger distance effect has been observed in children with DD in some studies (e.g., Mussolin et al., 2010). In contrast, a recent meta-analysis (Schwenk et al., 2017) reported that no qualitatively different distance effect in symbolic or non-symbolic comparison paradigms could be found in children with DD. Most results suggested a relatively more inefficient than a qualitatively different numerical processing pattern in DD (Landerl and Kölle, 2009; Kuhn et al., 2013; Landerl, 2013). Generally, the distance effect was observed for children with and without DD in symbolic and non-symbolic magnitude comparisons from 2nd grade onward (Landerl and Kölle, 2009; Landerl, 2013). Some studies reported that the distance effect was stable across development (Reeve et al., 2012; Landerl, 2013), while others reported a steadily decreasing distance effect with age (Holloway and Ansari, 2008; Moore and Ashcraft, 2015). DD children were generally more inefficient than TD children across the elementary school (Holloway and Ansari, 2009). The results did not suggest a qualitatively different processing pattern in DD; instead, they suggested a developmental delay. A systematic analysis investigating small and large distances for symbolic and non-symbolic magnitude comparisons between children with and without DD in the 2nd, 3rd, and 4th grades is still lacking.

For the less frequently used mixed comparison task, tapping several core systems of numerical processing (OTS, ANS, and visual-Arabic modules, respectively; Raddatz et al., 2017), there is currently a lack of knowledge. The task addresses children’s mapping skills in addition to their numerical processing skills, as in solving the mixed comparison task, two quantities in different modalities (e.g., point sets and Arabic numerals) are compared (Kuhn et al., 2013). To solve the mixed comparison task efficiently, children need to know how the non-symbolic and written Arabic numerals are related to each other (Kuhn et al., 2013). Previous results have shown that DD children were less efficient compared to TD children but did not seem to be disproportionately impaired (Kuhn et al., 2013). Research examining the influence of the age of children with and without DD in the context of this task is still lacking.

### 1.4 Number sets

The number sets task developed by Geary et al. (2009) primarily taps the visual-Arabic module (Raddatz et al., 2017). In this task, children compare an Arabic number (target) with a number set displayed below (Kuhn et al., 2013). The number set consists of two Arabic numbers, two different numerosities of dots, or combining an Arabic number and one numerosity of dots (Raddatz et al., 2017). Children decide whether the target matches the number of the total number set (e.g., target = 9, set 4 (Arabic number) + five dots) (Raddatz et al., 2017). Non-symbolic numerosities in this task can be in the subitizing or counting range, requiring ANS processing. In addition, numerosities from number symbols tap the visual-Arabic module. Although this task does not differentiate well between single hypotheses pertaining to the causes of DD, it significantly distinguishes between children with and without DD (see Geary et al., 2009). Previous studies showed that children with DD show impaired performance in this task compared to TD children (e.g., Kuhn et al., 2013; Raddatz et al., 2017; von Wirth et al., 2021). Brankaer et al. (2014) investigated similar mapping tasks and observed that mapping abilities continue to develop through primary school. Although some authors examined the performance of elementary school students with DD in this task (e.g., Kuhn et al., 2013), it is still unclear whether performance in children with DD develops qualitatively different from children without DD.

### 1.5 Number line estimation

The number line estimation task entails transcoding a numerical value into a spatial position on a visual line bounded by two numbers (e.g., 0 and 100) (Siegler et al., 2009). Number line tasks require an understanding of ordinality and estimation skills (see von Aster and Shalev, 2007), thus tapping the ANS (Feigenson et al., 2004). A less mature mental number line results in difficulties representing and distinguishing between numerosities. For example, younger children overrepresent small numbers on the number line (e.g., they locate 300 at about 450 on a number line from 0 to 1,000; Booth and Siegler, 2006). This logarithmic rather than linear conception of the mental number line leads to less accurate estimates (Booth and Siegler, 2006). In line with the defective ANS and AD hypotheses, children with DD should display problems locating numbers on the number line (see Wilson and Dehaene, 2007). Several empirical results support this assumption (Geary et al., 2008; Decarli et al., 2023). DD children’s estimations deviated more strongly from the target number and corresponded more closely to a logarithmic than a linear pattern. Generally, mental number line precision improved with age (Booth and Siegler, 2006; Landerl, 2013). However, Landerl’s (2013) findings indicated that DD children’s estimates were less accurate than the TD children’s estimates throughout elementary school. Nevertheless, only at the first measurement point were numbers represented in a logarithmically compressed way. The low accuracy of DD children’s performance supports the assumption of a persistent general inaccuracy, which, however, does not differ qualitatively from TD children. Whether DD children’s mental number line precision develops with a delay or whether it remains unspecified has remained unclear.

### 1.6 Current study

Most findings indicate delayed rather than qualitatively different numerical processing in dyscalculia. In line with extant research, we expected that DD children display deficits in basic numerical skills, suggesting a lower efficiency and accuracy of numerical processing rather than a qualitative difference (*disproportionate* impairment). Generally, an impairment is present when DD children perform significantly worse than TD children. In detail, we addressed the following two overarching research questions (RQs): (1) Are DD children qualitatively different in basic numerical skills compared to TD children, or is there, instead, a developmental delay for DD children? (2) Are deficits in the basic numerical skills most compatible with the ANS-, OTS-, and/or AD-deficit hypothesis?

We examined various basic numerical tasks in children with and without DD in a cross-sectional design. Based on the numerical development in the first school years, it would be conceivable that differences between children with and without DD are due to numerical age development. To control for this effect of age development, we contrasted children’s performance for three grades (2nd–4th grades). Concerning age-related mathematical development, we expected that children become increasingly efficient. The increasing efficiency in numerical processing should, however, vary depending on the task.

With regard to RQ1, we investigated whether there is evidence for qualitatively different numerical processing in children with DD that is (not) moderated by grade level, indicating qualitatively different numerical processing (*over-additive impairment*) in children with DD. If the deficits are moderated by grade level, this would suggest that the development of the DD children is delayed in view of the ability-level-match design.

Furthermore, inspired by the ability-level-match design, we compared the basic numerical profiles of 4th grade DD children with the performance of 2nd grade TD children to examine whether DD is a developmental delay or whether DD results in a qualitatively different numerical processing profile. The interval of two school years (similar to Skagerlund and Träff, 2014) was chosen as previous work showed that the developmental delay varies between 1 and 5 years depending on the task (e.g., Piazza et al., 2010). Assuming that children with DD are developmentally delayed, the following patterns of results were expected: (a) There is a developmental delay of more than 2 school years in children with DD. In this case, TD children in 2nd grade will be substantially more efficient in solving basic numerical tasks than the DD children in 4th grade. (b) The developmental delay of the DD children is less than 2 school years; the DD children in 4th grade will be significantly better than the TD children in 2nd grade. (c) DD children’s numerical profile indicates qualitatively different numerical processing, suggesting a disproportionately large impairment, indicated by an interaction of the factors group and tasks.

With regard to RQ2, we compared different basic numerical paradigms for children with and without DD. Given that AD holds, we expected that accessing numerosities from symbols would be disproportionately impaired compared to non-symbolic numerosities. For example, the pattern of the following results would be consistent with the AD hypothesis: in a magnitude comparison task, children with DD are substantially slower than TD children in comparing symbolic magnitudes (i.e., numbers), whereas the difference between DD and TD children is much smaller when comparing non-symbolic numbers (Rousselle and Noël, 2007). Based on the literature, we anticipated that children with DD would show longer RTs for magnitude comparisons but no qualitatively different distance effect. Thus, children with DD are expected to be more inefficient and less accurate in magnitude comparisons (indicated by an effect of the group), but their mental number line representation should not seem qualitatively abnormal. Thus, we expected no disproportionate impairment, as indicated by the absence of an interaction between group and task conditions (small vs. large magnitudes). Pertaining to the three hypotheses about the causes of DD (OTS-, ANS-, and AD-deficit hypothesis), we expected patterns of impairment in the following tasks (see Table 1).

**Table 1:** Expected pattern of impairments in line with the ANS deficit hypothesis, OTS deficit hypothesis, and AD hypothesis.

Tasks	ANS	OTS	AD
Dot enumeration subitizing range (1–3/1–4)	Not impaired	Impaired	Not impaired
Dot enumeration counting range (4–9/5–9)^2^	Impaired	Not impaired	Not impaired
Number comparison^1,2^	Impaired	Not impaired	Impaired
Mixed comparison^1,2^	Impaired	(Not impaired)*	Impaired
Dot magnitude comparison/Panamath^1^	Impaired	Not impaired	Not impaired
Number sets^3^	Impaired	(Impaired)*	Impaired
Number line estimation^1^	Impaired	Not impaired	Impaired

The study’s tasks substantially tap the analog magnitude,^1^ verbal-phonological,^2^ and visual-Arabic^3^ module of the Triple Code Model by Dehaene (1992). *Tasks cannot differentiate sharply between hypotheses because tasks substantially tap the ANS and visual-Arabic module, but points (tapping the ANS) can be in the subitizing (tapping OTS) or counting range (tapping verbal-phonological module). ANS deficit = general deficit in representing numerosities; OTS deficit = specific deficits in an object-based attentional system; AD = deficits in accessing numerosities from symbols (based on Andersson and Östergren, 2012).

## 2 Materials and methods

### 2.1 Participants and procedure

A total of *N* = 480 children (2nd–4th grade) composed of 68 DD (mathematical abilities, see instruments: *T*-score ≤ 38; IQ > 70) and 412 TD (mathematical abilities: *T*-score > 38; IQ > 70) were recruited from 46 classes in 8 primary schools in Germany. The local ethics committee approved the study protocol, and parental consent was obtained before testing. DD and TD samples included 47 and 78 children, respectively, with comorbid reading disorders (reading fluency, PR ≤ 16). We did not exclude these children because it has been shown that children with reading disorders were only selectively impaired in verbal number tasks (e.g., counting; Raddatz et al., 2017).

Gender was evenly distributed between the grades, χ^2^ (2) = 0.916, *p* = 0.633 and groups, χ^2^ (1) =3.392, *p* = 0.655. The detailed demographics of all subjects are summarized in Table 2.

**Table 2:** Demographic characteristics, scores on mathematical abilities, intelligence, and reading fluency by grade and mathematical ability.

	2nd grade		3rd grade		4th grade	
Details	TD	DD	TD	DD	TD	DD
*n* (boys)	177 (85)	17 (8)	180 (84)	34 (10)	55 (29)	17 (6)
	*M* (*SD*)	*M* (*SD*)	*M* (*SD*)	*M* (*SD*)	*M* (*SD*)	*M* (*SD*)
Age, in months	98.56_a_ (6.88)	98.62_a_ (10.93)	110.27_b_ (6.77)	111.12_b_ (6.45)	120.90_c_ (6.23)	120.93_c_ (9.03)
HRT 1–4	104.88_a_ (11.40)	77.38_b_ (3.66)	103.32_a_ (12.62)	75.44_b_ (5.43)	102.65_a_ (13.69)	77.13_b_ (5.07)
CFT^1^	103.20_a_ (13.04)	95.68_a,c_ (12.31)	104.29_a_ (12.03)	94.50_c_ (13.23)	96.44_b,c_ (13.59)	88.03_c_ (9.46)
SLS 1–4	97.51_a_ (15.94)	79.35_b_ (13.53)	99.72_a_ (14.56)	78.00_b_ (16.58)	101.00_a_ (18.71)	82.47_b_ (14.61)

1, NA for gender and 39 NA for age; HRT 1–4, Heidelberger Rechentest (mathematical abilities); CFT, Culture Fair Intelligence Test (intelligence, intelligence quotient); SLS 1–4, Salzburger Lese-Screening für die Klassenstufen 1–4 (reading fluency, reading quotient). Scaling for all measures (*M*: 100/*SD*: 15). *Post-hoc* comparisons between groups were based on Holm’s method (*p* < 0.05). Mean scores with the same indices (per row) do not differ significantly. ^1^ Culture Fair Intelligence Test 1–Revision (CFT 1-R) was used for 2nd and 3rd graders, and Culture Fair Intelligence Test 2–Revision (CFT 20-R) was used for 4th graders.

### 2.2 Instruments

Tasks measuring reading fluency, non-verbal intelligence, and mathematical abilities were administered in class, while tasks assessing basic numerical skills were computer-administered in smaller groups of 13 pupils.

#### 2.2.1 Arithmetic

Children’s mathematical abilities were examined by the Arithmetic Operations of the *Heidelberger Rechentest* (HRT 1–4; Engl.: Heidelberger Numeracy Test 1–4; Haffner et al., 2005). The paper-based speed test consists of two scales, which comprise a total of 11 subtests. The first scale Arithmetic Operations (test–retest reliability r_tt_ = 0.93; Haffner et al., 2005) includes the following subtests: addition (e.g., 3 + 5), subtraction (e.g., 5–3), multiplication (e.g., 2 · 3), division (e.g., 9 ÷ 3), complement task (e.g., __ + 3 = 10), and greater (>)/less (<)/equal (=) number comparisons (e.g., 3 __ 71); and the second scale Numerical-logical and Visual-spatial skills (r_tt_ = 0.87; Haffner et al., 2005) comprises: numerical sequences (e.g., 12 11 10 9 8 7_ _ _), length estimation, dice counting), counting objects/figures, and connecting numbers. Each subtest consists of at least 10 (e.g., connecting numbers) and at most 40 (e.g., addition) tasks arranged in order of increasing difficulty. The children had to solve as many tasks as possible within 2 min for each subtest. To calculate a total test score, both subscales were combined.

#### 2.2.2 Intelligence

The non-verbal intelligence of 2nd and 3rd graders was examined by three subtests of the *Grundintelligenztest Skala 1-Revision* (CFT 1-R; Engl.: Culture Fair Intelligence Test 1-R Scale 1; Weiß and Osterland, 2013): series completion, classification, and matrices (r_tt_ = 0.95). The 4th graders were assessed using four subtests of the *Grundintelligenztest Skala 2-Revision* (CFT 20-R; Engl.: Culture Fair Intelligence Test 20-R Scale 2; Weiß, 2006): series completion, classification, matrices, and topologies (r_tt_ = 0.80). The test score (IQ) was calculated from the subscales used in each case.

#### 2.2.3 Reading fluency

Reading fluency was measured using the paper-based *Salzburger Lese-Screening für die Klassenstufen 1*–*4* (SLS 1–4; Engl.: Salzburg reading screening test for grades 1–4; parallel-forms reliability = 0.90; Mayringer and Wimmer, 2003). The children were presented with a list of 70 simple sentences containing correct and incorrect statements (e.g., “bananas can talk”). Within 3 min, as many sentences as possible had to be read and judged with regard to their correctness. The test score depends on the total number of sentences judged correctly.

All tasks assessing basic numerical skills given below consisted of the *CODY-M 2–4 battery* (Kuhn et al., 2017), except the Panamath task.

#### 2.2.4 Dot enumeration (DE)

Children were presented with several black dots (1–9) and asked to count the dots as quickly as possible. We chose a data-driven approach to divide the number of points to be counted into the subitizing and counting range; based on the model fit of the piecewise regressions, we set the subitizing range for each child individually between 1 and 3 or 1 and 4. More than half of the children (*n* = 266) could subitize three items. Depending on the number of dots, the subitizing (1–3/1–4) or counting (4–9/5–9) skills are captured. Each magnitude 1 to 9 was presented twice (a total of 18 items; time limit: 2 min). Based on the total area of the points, no clear conclusions can be drawn about the quantity to be counted. The median of the children’s RTs, averaged over all correct answers and separately for the subitizing and the counting range, was used as the test score (Raddatz et al., 2017).

#### 2.2.5 Number comparison (NC)

Pairs of single-digit Arabic numbers were presented on a screen. The task was to compare the Arabic numbers and select the larger Arabic number as quickly as possible (27 items; lime limit: 1.5 min). Numerical distances between both stimuli (small: 1–3; large: 4–6) varied systematically between one and six. Each difference appeared four times in a random order, but the same for all participants (Raddatz et al., 2017). The median of the children’s RTs for correct answers for small (1–3) and large (4–6) distances were calculated (e.g., Holloway and Ansari, 2009; Kuhn et al., 2013).

#### 2.2.6 Mixed comparison (MC)

This task works similarly to number comparison, differing only in one quantity being represented as a cloud of one to nine dots, instead of a number. When creating the dot stimuli, care was taken to ensure that the overall area of the dots does not allow unambiguous conclusions to be drawn about the quantity to be counted (Kuhn et al., 2017). The test score was calculated in the same way as in the number comparison task. Together, these three tasks represent the basic numerical processing scale, which has good test–retest reliability, r_tt_ = 0.72 (Kuhn et al., 2017).

#### 2.2.7 Dot magnitude comparison (Panamath)

Children were presented with 48 items consisting of yellow and blue dots (ranging from 5 to 21) on the screen and had to decide as quickly as possible which point cloud contained more dots without counting them (e.g., Halberda et al., 2008). In line with Raddatz et al. (2017), the items were presented with four ratios between the two sets: 1.2, 1.4, 1.6, and 2.6 (12 items each). The total score of correct answers and the median of the children’s RTs for correct answers were calculated.

#### 2.2.8 Number sets test (NS)

The speed and accuracy in identifying and processing quantities in different representations were measured using a task based on Geary et al. (2009). Participants had to compare a number set (consisting of numerosities of dots and/or an Arabic number) shown at the bottom of the screen with a target (Arabic number) at the top of the screen, then they had to decide as quickly as possible whether the sum of the number set shown at the bottom was equal to the target shown above (Raddatz et al., 2017). The time limit for each task type (target number 5 or 9) was 1.5 min. To calculate the test score, the incorrect answers (false alarms) were subtracted from the correct answers (hits) (Kuhn et al., 2017).

#### 2.2.9 Number line estimation (NL)

The acuity/precision of the mental number line was measured using a task based on Siegler and Booth (2004). Participants were asked to locate a presented number (1–99) on a number line with endpoints 0 and 100. For the analyses, the mean deviation between the target number and the answer was calculated (Kuhn et al., 2017). The time limit per item (23 in total) was 3.5 min (Raddatz et al., 2017). The number sets test and number line estimation are part of the complex number processing scale, which has good test–retest reliability, r_tt_ = 0.76 (Kuhn et al., 2017).

### 2.3 Statistical analyses

All statistical calculations were performed using the statistical software R (version 4.3.1; R Core Team, 2023).

Our observations are cross-sectional clustered data: classes within schools (level 3), students within classes (level 2), and children’s performance in different tasks within students (level 1). We used a multilevel approach to account for the clustering.

First, intercept-only models with schools as fixed effects were specified separately for each task. Intraclass correlation coefficients (ICCs) were calculated. When a school was associated with children’s performance on basic numerical tasks, school was retained as a fixed effect in the model specification of linear mixed models (LMMs).

Second, random-intercept-constant-slope (rics) models were specified and tested separately for each task. In the rics models, we included the predictor’s group (typical/dyscalculic), grade (2/3/4), and task condition (e.g., dot enumeration: subitizing/counting) and their interactions as fixed effects, while we considered classes (grade: 2/3/4) and students as random intercepts. The covariates IQ and reading fluency were regarded as fixed factors. Only a two-level structure (classes within schools (level 2) and students within classes (level 1)) was used for number sets and the number line task, as no task conditions were clustered within children.

To account for heteroscedasticity, we used a bias-reduced linearization (BRL) generalization, which corrects cluster-robust variance estimation (CRVE) in conjunction with a Satterthwaite approximation for t-tests. The methods are implemented in an R package called clubSandwich (Pustejovsky and Tipton, 2018). Non-normal distributions are less critical for LMMs (Schielzeth et al., 2020).

Third, pairwise contrasts based on fitted models with Satterthwaite approximation for degrees of freedom using Holm’s method were calculated for all significant effects to identify the effects more precisely based on mean differences. When the results of the pairwise contrasts differed from the rics model, the results of the rics model were used because they are more robust.

An interaction of group × task (e.g., number comparison: small and large distances) in the rics model would provide evidence for qualitatively different numerical processing (over-additive impairment) in children with DD. A significant effect of group and the absence of the above interaction for a given dependent variable (basic numerical task) would indicate a less efficient but not qualitatively different numerical processing (additive impairment) in children with DD. An interaction of group × grade would suggest a difference in numerical development between children with and without DD. If qualitative differences between children with and without DD are moderated by grade level, this would be shown in an interaction of group × task × grade, indicating that the qualitative differences between groups are not constant across grades. Whether interactions with the grade level indicate a qualitatively different developmental trajectory or rather a developmental delay needs to be tested with pairwise contrasts.

If the basic numerical skills of children with DD develop with a delay, children with DD would have to reach a comparable level of numerical processing as the TD children later. Therefore, we examined whether DD children in 4th grade catch up in basic numerical skills with TD children in 2nd grade or whether there is evidence of persistent inefficiency or qualitative differences in numerical processing. A significant effect of group (TD2/DD4) indicates a developmental delay across basic numerical skills, whereas an interaction of group × task indicates a qualitative difference in numerical processing.

## 3 Results

### 3.1 Dot enumeration

**Figure 2:**
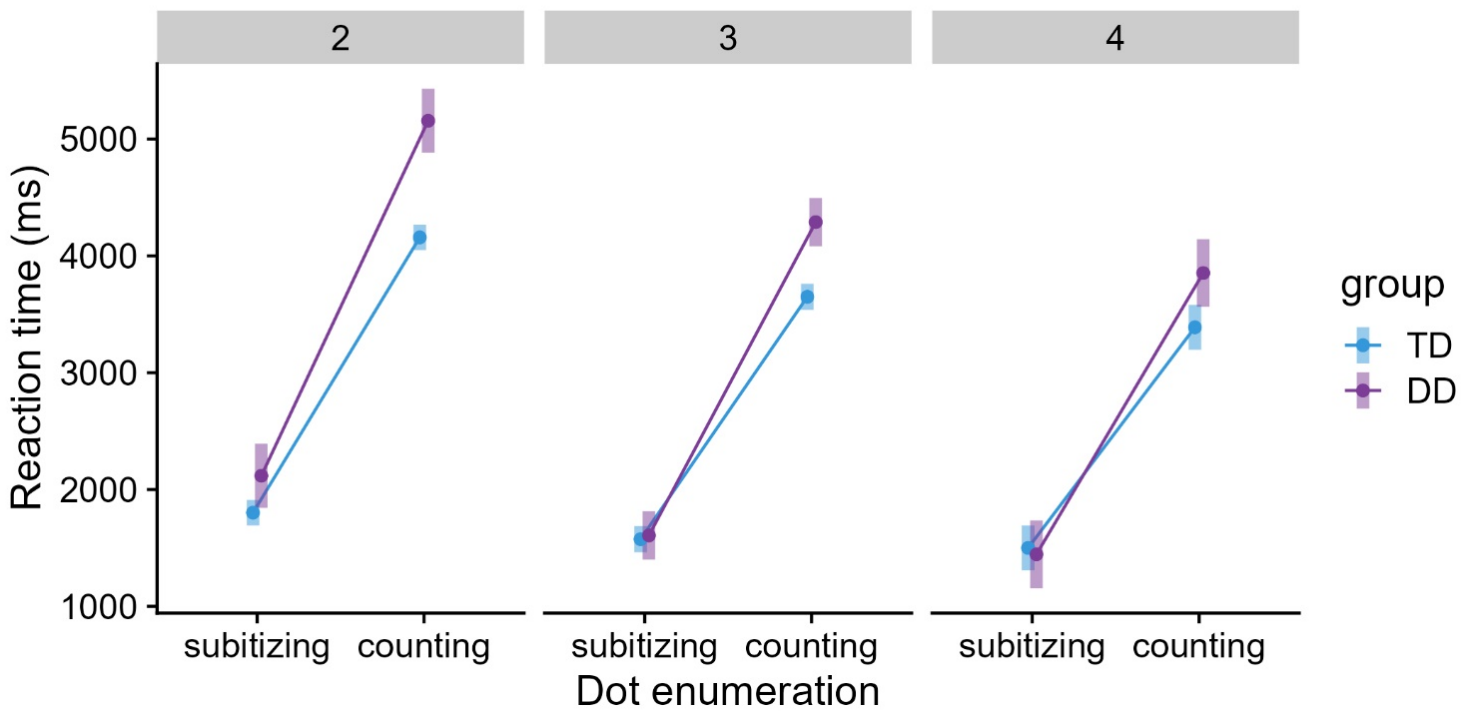
Means and 95% confidence intervals (CIs) for dot enumeration grouped by group and grade.

The predictors group (typical/dyscalculic), grade (2/3/4), and dot enumeration (subitizing/counting) and their interactions, except dot enumeration × group × grade, were significant (see Table 3). Pairwise contrasts based on the rics model showed that the DD group required significantly longer RTs than the TD group, *p* < 0.0001. RTs decreased systematically across grades (g2 > g3 > g4, *ps* < 0.05). Point sets in the counting range (4–9/5–9) resulted in longer RTs than point sets in the subitizing range (1–3/1–4), *p* < 0.0001. The large mean difference between the DD and TD children for the counting range (see Figure 2) resulted in a significant interaction of dot enumeration × group. The DD group required significantly longer RTs than the TD group in the counting range (*p* < 0.0001); however, not in the subitizing range (*p* = 0.235). The interaction of dot enumeration × grade may have been related to a relatively small decrease in RTs from 3rd to 4th grade for both task conditions, especially for the subitizing range (*ps* < 0.01, except 3rd vs. 4th grade for subitizing). The group × grade interaction showed that children with and without DD differed significantly in 2nd and 3rd grade (*p* < 0.01) but not in 4th grade (*p* = 0.393). Grade level trends within groups indicated that DD and TD children reached a stable level in 3rd grade (TD2 > TD3 = TD4; DD2 > DD3 = DD4, *ps* < 0.0001). In addition, higher levels of reading fluency were associated with lower RTs on the dot enumeration task, but the effect of IQ was not significant.

**Table 3:** Results of the linear mixed model using task condition, group, grade, reading fluency, and intelligence to predict children’s performance on dot enumeration.

Predictors	*B*	Robust S.E.	*t*	*df*	*p*
(Intercept)	2829.701	185.953	15.217	28.9	< 0.001
DE^a^	2357.316	61.734	38.185	14.0	< 0.001
Group^b^	316.240	133.730	2.365	8.9	0.043
Grade^c^ 3	−227.064	54.752	−4.147	32.7	< 0.001
Grade 4	−301.789	63.290	−4.768	8.7	0.001
Reading fluency	−8.004	1.285	−6.228	26.3	< 0.001
IQ	−2.549	2.074	−1.229	29.6	0.229
DE × group	682.125	185.825	3.671	8.5	0.006
DE × grade 3	−281.861	75.630	−3.727	26.6	0.001
DE × grade 4	−468.362	121.968	−3.840	6.9	0.007
Group × grade 3	−284.101	154.505	−1.839	16.4	0.084
Group × grade 4	−370.736	138.137	−2.684	9.9	0.023
DE × group × grade 3	−74.698	229.762	−0.325	16.2	0.749
DE × group × grade 4	−162.668	293.630	−0.554	10.1	0.592
Random effects					
τ_00 IDstudent: IDclass_	290.73				
τ_00 IDclass_	154.83				
*δ* ^2^	453.10				
ICC _IDstudent: IDclass_	0.297				
ICC _IDclass_	0.141				

The model summary based on *p*-values for fixed effects was calculated using Satterthwaite approximations.

DE = Dot enumeration. ^a^Subitizing is the reference. ^b^Typical developed is the reference. ^c^Grade 2 is the reference.

### 3.2 Number comparison

**Figure 3:**
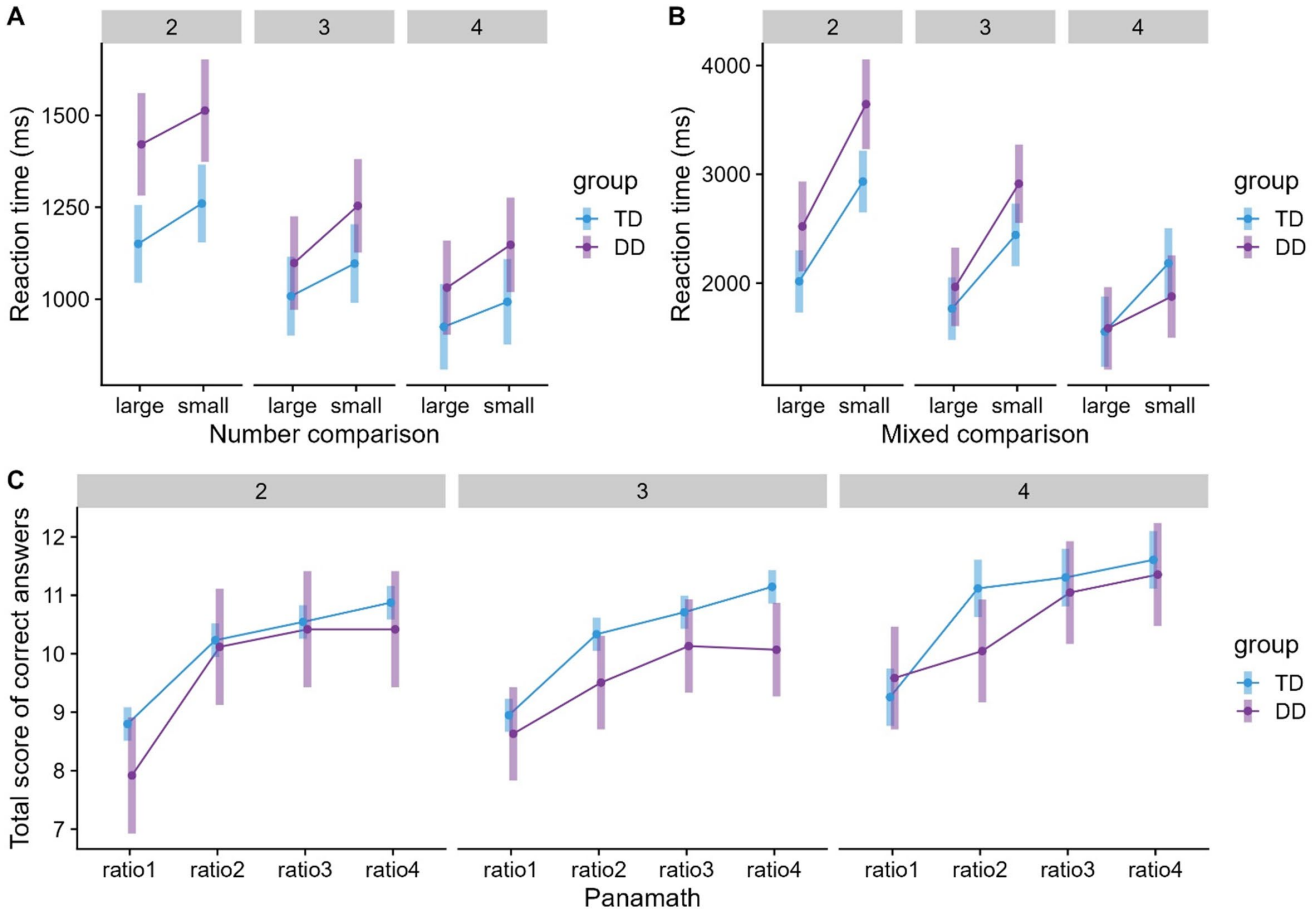
Means and 95% confidence intervals (CIs) for **(A)** number comparison, **(B)** mixed comparison and **(C)** Panamath grouped by group and grade. Ratio 1 = 1.2; ratio 2 = 1.4; ratio 3 = 1.6; and ratio 4 = 2.6 (12 items each).

The predictors group (typical/dyscalculic), grade (2/3/4), and number comparisons (large/small distances) were significant (see Table 4). Subsequent pairwise comparisons based on the rics model showed that DD children required significantly longer RTs than TD children (*p* < 0.0001). RTs decreased across grades (g2 > g3, *p* < 0.05, except g4 vs. g3). As expected, smaller numerical distances led to longer RT (*p* < 0.0001) (see Figure 3A). No interactions were found. Using reading fluency as a fixed effect showed that higher reading fluency was associated with lower RTs on the number comparison task, but the effect of IQ was not significant. A fixed effect of the school indicated that students from school A required longer RTs relative to school H.

**Table 4:** Results of the linear mixed model using task condition, group, grade, reading fluency, and intelligence to predict children’s performance on number comparison.

Predictors	*B*	Robust S.E.	*t*	*df*	*p*
(Intercept)	1253.521	82.264	15.238	28.7	< 0.001
NC^a^	109.712	11.386	9.636	14.0	< 0.001
Group^b^	270.653	79.434	3.407	9.0	0.008
Grade^c^ 3	−142.202	33.080	−4.299	33.0	< 0.001
Grade 4	−225.639	35.220	−6.407	8.5	< 0.001
Reading fluency	−1.894	0.457	−4.144	26.3	< 0.001
IQ	−0.380	0.787	−0.484	29.7	0.632
School A ^d^	235.377	33.430	7.041	4.9	0.001
NC × group	−17.888	60.545	−0.295	8.5	0.775
NC × grade 3	−21.079	14.617	−1.442	26.6	0.161
NC × grade 4	−41.612	19.944	−2.086	6.9	0.076
Group × grade 3	−180.461	88.724	−2.034	16.4	0.058
Group × grade 4	−164.044	83.246	−1.971	9.5	0.079
NC × group × grade 3	84.623	68.657	1.233	16.2	0.235
NC × group × grade 4	66.230	74.782	0.886	10.1	0.396
Random effects					
τ_00 IDstudent: IDclass_	186.59				
τ_00 IDclass_	63.26				
*δ* ^2^	90.74				
ICC _IDstudent: IDclass_	0.678				
ICC _IDclass_	0.180				

The model summary based on *p*-values for fixed effects was calculated using Satterthwaite approximations. NC = number comparison.

^a^Large distance is the reference. ^b^Typical developed is the reference. ^c^Grade 2 is the reference. ^d^School H is the reference.

### 3.3 Mixed comparison

The predictors group (typical/dyscalculic), grade (2/3/4), and mixed comparison (small/large distances) were significant. Subsequent pairwise comparisons based on the rics model revealed longer RTs for DD than TD children (*p* < 0.01), a systematic decrease in RTs across grades (g2 > g3 > g4, *ps* < 0.001), and longer RTs for small than for large distances (*p* < 0.001). Figure 3B shows that children in all grades needed longer RTs for small distances, especially in 2nd grade. This may have resulted in the mixed comparison × grade interaction. The pairwise comparisons showed that children’s RTs decreased across grades when comparing the small distances (*ps* <. 001). In contrast, there was only a significant difference between 2nd and 3rd graders (*p* <. 001) for large distances. We found no evidence of a mixed comparison × group effect (see Table 5). However, the large difference between children with and without DD for small distances in 2nd grade (see Figure 3B) may have resulted in a significant interaction of mixed comparison × group × grade. Subsequent comparisons showed that in 2nd and 3rd grades, DD children needed longer RTs in small distances compared to TD children (*ps* < 0.05). At large distances, children with and without DD did not differ significantly (*ps* > 0.05). Grade level trends within groups showed that RTs of DD children for large distances decreased from 2nd to 3rd grade (DD2 > DD3 = DD4, *p* < 0.01), but there was no significant development for TD children (TD2 = TD3 = TD4, *ps* > 0.05). In small distances, TD children reached a stable level as early as 3rd grade (TD2 > TD3 = TD4, *p* < 0.001). In contrast, DD children continued to develop until 4th grade (DD2 > DD3 > DD4, *ps* < 0.05). Using reading fluency and IQ as fixed effects, higher IQ was associated with lower RTs in the mixed comparison task. The effect of reading fluency was not significant. In addition, it was found that belonging to school A was associated with poorer performance compared to school H (see Table 5).

**Table 5:** Results of the linear mixed model using task condition, group, grade, reading fluency, and intelligence to predict children’s performance on mixed comparison.

Predictors	*B*	Robust S.E.	*t*	*df*	*p*
(Intercept)	2400.484	192.238	12.487	28.4	< 0.001
MC^a^	916.887	72.139	12.710	14.0	< 0.001
Group^b^	504.641	239.509	2.107	9.0	0.065
Grade^c^ 3	−250.340	58.506	−4.279	30.6	< 0.001
Grade 4	−462.388	74.686	−6.191	8.1	< 0.001
Reading fluency	−1.325	2.141	−0.619	26.3	0.541
IQ	−5.489	1.963	−2.797	30.2	0.009
School A^d^	599.333	76.148	7.871	4.6	0.001
MC × group	206.172	193.653	1.065	8.5	0.316
MC × grade 3	−240.143	85.361	−2.813	26.6	0.009
MC × grade 4	−288.414	85.098	−3.389	6.9	0.012
Group × grade 3	−305.458	276.649	−1.104	16.3	0.286
Group × grade 4	−474.660	267.062	−1.777	9.6	0.107
MC × group × grade 3	65.113	232.200	0.280	16.2	0.783
MC × group × grade 4	−541.909	230.088	−2.355	10.1	0.040
Random effects					
τ_00 IDstudent: IDclass_	490.44				
τ_00 IDclass_	138.33				
*δ* ^2^	471.95				
ICC _IDstudent: IDclass_	0.483				
ICC _IDclass_	0.100				

The model summary based on p-values for fixed effects was calculated using Satterthwaite approximations. MC = mixed comparison.

^a^Large distance is the reference. ^b^Typical developed is the reference. ^c^Grade 2 is the reference. ^d^School H is the reference.

### 3.4 Dot magnitude comparison (Panamath)

Not all children completed this task due to a computer technical problem. Data for the Panamath task were only available for *n* = 322 children (*n* = 39 with DD). TD children’s total score was not significantly higher than DD children’s score (see Table 6). The predictor Panamath (ratios: 1.2/1.4/1.6/2.4) was significant. Subsequent pairwise comparisons based on the rics model showed that the total test score of correct answers decreased systematically over the ratios (r4 > r3 > r2 > r1, *ps* < 0.01, except r3 vs. r4). Furthermore, an interaction of Panamath × group × grade was found. Pairwise comparisons focused on group differences within a grade level and condition (ratio) revealed no significant differences between TD and DD children. However, children’s total test scores of correct answers increased from ratio 1 to ratio 2 (also see Figure 3C). While this was evident for TD children in all grades, for DD children, the effect was only found in 2nd grade (*ps* < 0.01). Reading fluency and IQ were not associated with the Panamath task (see Table 6).

**Table 6:** Results of the linear mixed model using task condition, group, grade, reading fluency, and intelligence to predict children’s performance on Panamath.

Predictors	*B*	Robust S.E.	*t*	*df*	*p*
(Intercept)	7.323	0.587	12.471	16.8	< 0.001
Ratio 2^a^	1.432	0.162	8.838	7.4	< 0.001
Ratio 3	1.746	0.144	12.114	7.4	< 0.001
Ratio 4	2.076	0.142	14.671	7.4	< 0.001
Group^b^	−0.881	0.386	−2.281	6.8	0.058
Grade^c^ 3	0.150	0.230	0.652	15.4	0.524
Grade 4	0.459	0.301	1.523	5.9	0.180
Reading fluency	0.003	0.003	0.810	15.8	0.430
IQ	0.012	0.006	2.076	19.3	0.052
Ratio 2 × group	0.768	0.428	1.795	6.5	0.119
Ratio 3 × group	0.754	0.465	1.623	6.5	0.152
Ratio 4 × group	0.424	0.787	0.539	6.5	0.608
Ratio 2 × grade 3	−0.047	0.254	−0.185	15.5	0.856
Ratio 3 × grade 3	0.017	0.204	0.081	15.5	0.937
Ratio 4 × grade 3	0.120	0.222	0.542	15.5	0.596
Ratio 2 × grade 4	0.428	0.335	1.277	5.1	0.257
Ratio 3 × grade 4	0.301	0.280	1.074	5.1	0.331
Ratio 4 × grade 4	0.273	0.284	0.961	5.1	0.380
Group × grade 3	0.563	0.760	0.741	12.3	0.472
Group × grade 4	1.208	0.679	1.779	7.8	0.114
Ratio 2 × group × grade 3	−1.278	0.591	−2.162	12.3	0.051
Ratio 3 × group × grade 3	−1.017	0.510	−1.993	12.3	0.069
Ratio 4 × group × grade 3	−1.183	0.974	−1.215	12.3	0.247
Ratio 2 × group × grade 4	−2.167	0.888	−2.441	7.8	0.041
Ratio 3 × group × grade 4	−1.339	0.773	−1.732	7.8	0.123
Ratio 4 × group × grade 4	−1.003	0.948	−1.059	7.8	0.321
Random effects					
τ_00 IDstudent: IDclass_	1.15				
τ_00 IDclass_	0.00				
*δ* ^2^	1.09				
ICC _IDstudent: IDclass_	0.534				
ICC _IDclass_	0.006				

The model summary based on *p*-values for fixed effects was calculated using Satterthwaite approximations. ^a^Ratio 1 is the reference. ^b^Typical developed is the reference. ^c^Grade 2 is the reference.

### 3.5 Dot magnitude (non-symbolic) vs. number (symbolic) comparison

The predictors grade (2/3/4), distance (small/large), and task (non-symbolic/symbolic) were significant. Subsequent pairwise comparisons based on the rics model indicated that RTs decreased from 2nd to 4th grade (*p* < 0.0001) but not systematically across grades. There was no significant difference between 2nd and 3rd grade (see Table 7). Small distances resulted in longer RTs than large distances (*p*  < 0.0001) and symbolic tasks resulted in longer Rts than non-symbolic tasks (*p*  < 0.01). The interaction of task × distance showed that children required longer RTs for small distances than for large distances in both task conditions (*ps* < 0.01). Additionally, large distances in symbolic comparisons resulted in higher RTs than large distances in non-symbolic comparisons (*p* < 0.01). In contrast, the mean difference between small distances in symbolic comparisons and small distances in non-symbolic comparisons was not significant (*p* = 0.631). The task × group interaction showed that DD children required substantially longer RTs to compare symbolic and non-symbolic tasks than the TD children (*ps* < 0.05). Furthermore, within-group effects showed that in contrast to TD children, DD children did not need longer RTs for symbolic than non-symbolic tasks. Subsequent pairwise comparisons of the task × group × grade interaction, focusing on group differences within a grade level and a task condition, revealed that children with and without DD did not differ in any grade level. Grade-level trends within groups showed that there was no significant development in non-symbolic and symbolic tasks for DD and TD children. With one exception: TD children’s RTs for symbolic tasks decreased from 2nd to 3rd grade (*ps* < 0.01). Furthermore, DD and TD children substantially needed longer RTs to compare symbolic than non-symbolic tasks in 2nd grade (*ps* < 0.01), but there were no significant differences in 3rd or 4th grade (*p* = 1.000). When rerunning analyses without the 2nd graders, the task × group effect vanished. No significant effects were found for the covariates, but children from school A showed poorer performance in comparison to children from school H (see Table 7).

**Table 7:** Results of the linear mixed model using task conditions, group, grade, reading fluency, and intelligence to predict children’s performance on dot magnitude and number comparisons.

Predictors	*B*	Robust S.E.	*t*	*df*	*p*
(Intercept)	1137.349	138.610	8.205	17.0	< 0.001
Task^a^	155.792	41.326	3.770	7.4	0.006
Distance^b^	214.475	23.899	8.974	7.4	< 0.001
Group^c^	−4.594	74.967	−0.061	6.7	0.953
Grade 3^d^	−93.850	52.314	−1.794	16.0	0.092
Grade 4	−203.681	60.157	−3.386	5.8	0.016
Reading fluency	−0.551	0.812	−0.678	16.0	0.507
IQ	−1.411	1.315	−1.073	19.2	0.296
School A^e^	176.186	46.803	3.764	3.4	0.026
Task × distance	−111.449	29.202	−3.816	7.4	0.006
Task × group	254.008	85.669	2.965	6.5	0.023
Distance × group	43.225	144.160	0.300	6.5	0.774
Task × grade 3	−82.182	56.917	−1.444	15.5	0.169
Task × grade 4	−107.374	42.616	−2.520	5.1	0.053
Distance × grade 3	−60.671	29.796	−2.036	15.5	0.059
Distance × grade 4	−59.475	25.645	−2.319	5.1	0.068
Group × grade 3	279.338	280.175	0.997	12.2	0.338
Group × grade 4	110.484	123.294	0.896	7.6	0.398
Task × distance × group	−16.901	168.704	−0.100	6.5	0.923
Task × distance × grade 3	54.146	35.177	1.539	15.5	0.144
Task × distance × grade 4	26.821	33.322	0.805	5.1	0.457
Task × group × grade 3	−449.712	268.516	−1.675	12.3	0.119
Task × group × grade 4	−228.849	92.204	−2.482	7.8	0.039
Distance × group × grade 3	−129.560	165.147	−0.785	12.3	0.448
Distance × group × grade 4	146.159	151.149	0.967	7.8	0.363
Task × distance × group × grade 3	145.173	187.632	0.774	12.3	0.454
Task × distance × group × grade 4	−129.125	196.356	−0.658	7.8	0.530
Random effects					
τ_00 IDstudent: IDclass_	228.17				
τ_00 IDclass_	30.48				
*δ* ^2^	271.79				
ICC _IDstudent: IDclass_	0.396				
ICC _IDclass_	0.057				

The model summary based on p-values for fixed effects was calculated using Satterthwaite approximations. ^a^Non-symbolic is the reference. ^b^Large distance is the reference. ^c^Typical developed is the reference. ^d^Grade 2 is the reference. ^e^School H is the reference.

### 3.6 Number sets

**Figure 4:**
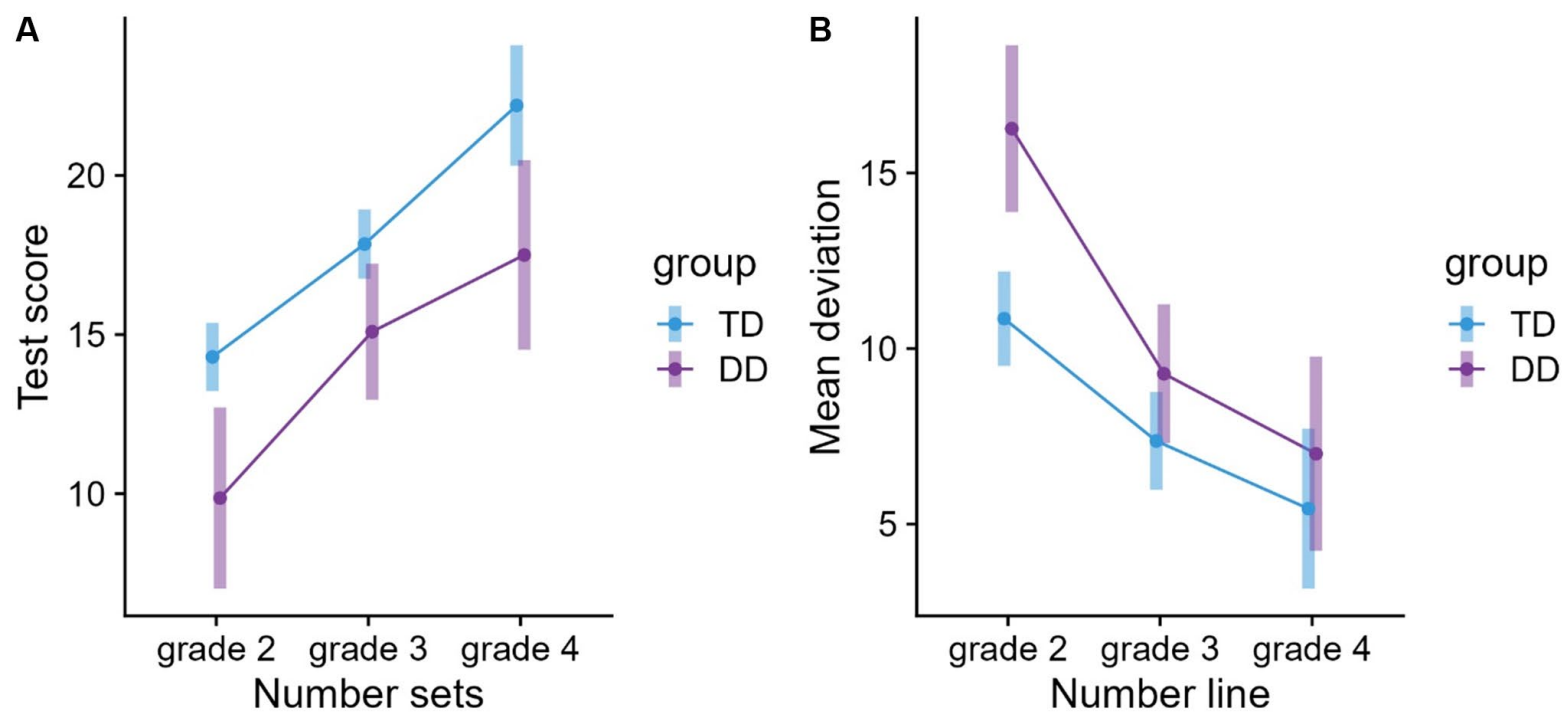
Means and 95% confidence intervals (CIs) for **(A)** number sets and **(B)** number line estimation grouped by grade and group.

The predictors group (typical/dyscalculic) and grade (2/3/4) were significant. Subsequent pairwise comparisons based on the rics model indicated a better test score for the TD than DD children (*p* < 0.0001), and an improvement across grades (g2 > g3 > g4, *ps* < 0.01, also see Figure 4A). Using reading fluency and IQ as fixed effects, both were significant. A higher level of IQ and reading fluency was associated with a better test score (see Table 8).

**Table 8:** Results of the linear mixed model using group, grade, reading fluency, and intelligence to predict children’s performance on number sets.

Predictors	*B*	Robust S.E.	*t*	*df*	*p*
(Intercept)	−8.391	2.844	−2.951	28.4	0.006
Group^a^	−4.433	1.614	−2.746	9.1	0.022
Grade 3^b^	3.547	0.746	4.757	31.4	< 0.001
Grade 4	7.900	0.776	10.177	8.5	< 0.001
Reading fluency	0.104	0.018	5.876	26.3	< 0.001
IQ	0.125	0.022	5.633	30.2	< 0.001
Group × grade 3	1.680	1.976	0.850	16.4	0.407
Group × grade 4	−0.264	2.857	−0.092	9.9	0.928
Random effects					
τ_00 IDclass_	1.31				
*δ* ^2^	5.62				
ICC _IDclass_	0.179				

The model summary based on *p*-values for fixed effects was calculated using Satterthwaite approximations. ^a^Typical developed is the reference. ^b^Grade 2 is the reference.

### 3.7 Number line estimation

The predictors group (typical/dyscalculic) and grade (2/3/4) were significant. Subsequent pairwise comparisons based on the rics model indicated a larger average deviation in the DD group than for the TD group (*p* < 0.0001), and a decrease in average deviation across grades (g2 > g3, g4, except g3 vs. g4, *ps* < 0.001). Figure 4B shows that especially DD children in 2nd grade were less accurate. A relatively small decrease in the mean deviations from 3rd to 4th grade resulted in a group × grade interaction. Pairwise comparisons of the interaction showed that the precision of arranging the digit on the number line increased within groups, but both groups reached a stable level as early as 3rd grade. Furthermore, the group × grade interaction revealed that only DD children in 2nd grade were less accurate than TD children (*p* < 0.001). There were no differences between the two groups in the 3rd and 4th grades (*ps* > 0.05). Further fixed effects of reading fluency and IQ were significant. A higher level of IQ and reading fluency was associated with a better test score (see Table 9).

**Table 9:** Results of the linear mixed model using group, grade, reading fluency, and intelligence to predict children’s performance on number line.

Predictors	*B*	Robust S.E.	*t*	*df*	*p*
(Intercept)	24.266	3.040	7.981	29.6	< 0.001
Group^a^	5.412	1.279	4.232	9.0	0.002
Grade 3^b^	−3.484	1.012	−3.443	36.4	0.001
Grade 4	−5.413	0.885	−6.114	9.7	< 0.001
Reading fluency	−0.048	0.016	−3.056	25.9	0.005
IQ	−0.087	0.018	−4.798	28.4	< 0.001
Group × grade 3	−3.496	1.440	−2.428	16.5	0.027
Group × grade 4	−3.847	1.722	−2.234	9.6	0.051
Random effects					
τ_00 IDclass_	2.58				
*δ* ^2^	4.11				
ICC _IDclass_	0.380				

The model summary based on p-values for fixed effects was calculated using Satterthwaite approximations. ^a^Typical developed is the reference. ^b^Grade 2 is the reference.

### 3.8 DD in 4th grade vs. TD in 2nd grade

For the profile analyses, the variables were z-standardized. Accordingly, scale differences between the tasks largely disappeared (Table 10). The predictor group (typical/dyscalculic) was significant. Subsequent pairwise comparisons based on the rics model showed that on average (across tasks), the TD children from the 2nd grade performed substantially worse than DD children in the 4th grade. No interaction between subgroup and grade was found, indicating a delayed basic numerical profile in DD children.

**Table 10:** Results of the linear mixed model using task condition, group, grade, reading fluency, and intelligence to predict children’s performance on all basic numerical tasks.

Predictors	*B*	Robust S.E.	*t*	*df*	*p*
(Intercept)	0.233	0.401	0.580	10.6	0.574
DE counting^a^	0.375	0.233	1.609	3.6	0.190
NC	0.482	0.183	2.636	3.6	0.064
MC	−0.020	0.191	−0.105	3.6	0.922
NS	0.339	0.394	0.861	3.2	0.448
NL	−0.019	0.515	−0.038	3.6	0.972
Panamath	0.280	0.429	0.651	3.6	0.554
Group^b^	0.737	0.271	2.721	4.7	0.045
Reading fluency	−0.004	0.002	−2.102	10.7	0.060
IQ	−0.003	0.003	−0.937	12.3	0.367
DE counting^a^ × group	−0.470	0.240	−1.960	4.3	0.116
NC × group	−0.501	0.193	−2.600	4.3	0.056
MC × group	−0.067	0.222	−0.300	4.3	0.778
NS × group	−0.854	0.419	−2.036	3.9	0.113
NL × group	−0.591	0.536	−1.104	4.3	0.327
Panamath × group	−0.326	0.461	−0.708	4.3	0.515
Random effects					
τ_00 IDstudent: IDclass_	0.14				
τ_00 IDclass_	0.23				
*δ* ^2^	0.92				
ICC _IDstudent: IDclass_	0.025				
ICC _IDclass_	0.043				

The model summary based on *p*-values for fixed effects was calculated using Satterthwaite approximations. NC = number comparison; MC = mixed comparison; NS = number sets; NL = number line; Panamath, median of the children’s RTs for correct answers.

^a^Subitizing is the reference. ^b^Dyscalculic grade 4 is the reference.

## 4 Discussion

We investigated whether DD children’s basic numerical skills are qualitatively different compared to TD or, instead, if there is a developmental delay for DD children (RQ 1). To answer this question, we investigated a wide range of basic numerical paradigms and compared basic numerical profiles of DD children in 4th grade with TD children in 2nd grade. Finally, competing hypotheses on the causes of DD were compared and it was examined whether these assumptions are stable across different grades (RQ 2). Because substantial mathematical development can be observed in the first years of schooling, we considered the children’s grade level (2–4) as a possible influencing factor. Thereby, we ruled out that differences between children with and without DD were due to mathematical age development.

Regarding the first RQ, DD children consistently displayed deficits in core markers of numeracy, with the exception of subitizing and dot magnitude comparison. DD children’s difficulties were manifested in lower efficiency and/or accuracy. Apart from counting, we found no evidence that DD children’s basic numerical skills were qualitatively different from those of TD children. Disproportionate impairments in processing numerosities from symbols and the qualitatively different distance effect in the mixed comparison task were moderated by grade level. The results suggest that children with DD exhibit developmentally delayed rather than qualitatively different numerical processing. Additionally, DD children showed a different developmental trajectory for mixed comparison, dot magnitude vs. number comparison, and number line, but in line with the profile analysis, this can be interpreted as a developmental delay. Overall, the results provided evidence for delayed numerical processing in DD.

The results regarding the question of the causes of DD supported the defective ANS rather than the AD hypothesis (see Table 11). We found no evidence for the OTS deficit hypothesis. These results appeared consistent across grades. In the following, we address the results pertaining to each task in detail.

**Table 11:** Results of impairments in line with the ANS deficit hypothesis, OTS deficit hypothesis, and AD hypothesis.

Tasks	ANS	OTS	AD	Results
Dot enumeration subitizing range (1–3 / 1–4)	Not impaired	Impaired	Not impaired	Not impaired
Dot enumeration counting range (4–9 / 5–9)^2^	Impaired	Not impaired	Not impaired	Qualitative impaired
Number comparison^1,2^	Impaired	Not impaired	Impaired	Delayed impaired
Mixed comparison^1,2^	Impaired	(Not impaired)*	Impaired	Delayed impaired
Dot magnitude comparison/Panamath^1^	Impaired	Not impaired	Not impaired	Not impaired
Number sets^3^	Impaired	(Impaired)*	Impaired	Delayed impaired
Number line estimation^1^	Impaired	Not impaired	Impaired	Delayed impaired

The study’s tasks substantially tap the analog magnitude,^1^ verbal-phonological,^2^ and visual-Arabic^3^ module of the Triple Code Model by Dehaene (1992). *Tasks cannot differentiate sharply between hypotheses because tasks substantially tap the ANS and visual-Arabic module, but points (tapping the ANS) can be in the subitizing (tapping OTS) or counting range (tapping verbal-phonological module). ANS deficit, general deficit in representing numerosities; OTS deficit, specific deficits in an object-based attentional system; AD, deficits in accessing numerosities from symbols (based on Andersson and Östergren, 2012).

### 4.1 Dot enumeration

The results confirmed prior studies (e.g., Andersson and Östergren, 2012; Decarli et al., 2020, 2023): Larger point sets led to longer RTs, supporting a qualitative difference in the perception and representation of large vs. small quantities. In contrast to previous studies (e.g., Kuhn et al., 2013; Decarli et al., 2023) and the OTS defective hypothesis, we found no evidence of a deficit in the subitizing range (similar to Skagerlund and Träff, 2014). The present results suggest, in line with the defective ANS hypothesis, that DD children’s counting skills were disproportionately impaired. This is unusual because most studies showed that children with DD were additively or over-additively impaired in subitizing (e.g., Landerl, 2013). However, we implemented a data-driven approach to determine the subitizing range. Other authors (e.g., Kuhn et al., 2013) set the subitizing range at three dots. Due to the variability in the subitizing range, it is possible that some studies have mixed the counting and subitizing skills, resulting in lower RTs in the counting range. We found no evidence that DD children’s over-additive impairment in the counting range was moderated by age development (grade level). Nevertheless, the disproportionately impaired counting skills in DD must be interpreted with some reservations because prior studies that found no evidence of a qualitatively different approach to counting did not include children with comorbid reading disorders (e.g., Schleifer and Landerl, 2011; Kuhn et al., 2013). In our study, a higher level of reading fluency was associated with lower RTs. Thus, verbal problems could cause a deficit in counting efficiency (see Schleifer and Landerl, 2011). Averaged across all items 1–9 (not differentiating between the counting and subitizing range), the differences between children with and without DD were moderated by grade level. Children with DD performed poorer than TD children in the 2nd and 3rd grade. However, in the 4th grade, there were no longer substantial group differences. Grade level trends within both groups reached a stable level in the 3rd grade, suggesting that DD children catch up and are merely developmentally delayed. Our results showed that counting skills and subitizing developed differently (similar to Schleifer and Landerl, 2011). Complementary to the findings of Schleifer and Landerl (2011), a stable competence level in subitizing was achieved in the 3rd grade, thus earlier than in counting. All in all, the results showed disproportionately impaired counting skills in DD. Future studies should determine the subitizing and counting range data-driven and consider the influence of reading-related difficulties.

### 4.2 Number comparison

The current results revealed that smaller numerical distances resulted in longer RTs, and DD children performed poorer than TD children but showed no greater distance effect than TD children (similar to Holloway and Ansari, 2008; Decarli et al., 2020, 2023). In line with Landerl and Kölle (2009), children with and without DD became more efficient across grades, but there was no evidence that the distance effect was moderated by grade level (similar to Holloway and Ansari, 2009; Landerl and Kölle, 2009; Reeve et al., 2012), or that the developmental trajectory of children with DD differed from that of TD children. As deficits in symbolic numerical processing are associated with an impaired ANS and AD, number comparisons cannot clearly discriminate between the hypotheses. However, it is certain that, for DD children tapping the visual-Arabic module causes problems. Although we found a fixed effect for reading fluency, we do not assume that our result was significantly confounded by this covariate because studies (e.g., Decarli et al., 2023) showed that children with DD and combined impaired children did not differ.

### 4.3 Mixed comparison

As expected, smaller distances resulted in longer RTs. This distance effect was evident in all grades. A stable level of competence was reached earlier in large distances than in small distances. Similar to the results of Kuhn et al. (2013), DD children performed worse than TD children. Children with and without DD did not differ in large distances, but DD children required longer RTs in small distances in the 2nd and 3rd grades. At first glance, a larger distance effect was observed in children with DD, in line with the ANS deficit (similar to Mussolin et al., 2010). However, there was no significant difference between DD and TD in small distances in the 4th grade. Furthermore, no interaction between mixed comparison and group was found. Thus, the result implies that children’s grade level moderates the distance effect, indicating that DD children’s performance is developmentally delayed. The result contradicts other studies that found no over-additive distance or age effects (see Schwenk et al., 2017). However, the finding may be related to the specificity of the task condition. Most studies compared symbolic or non-symbolic tasks, but not a mixture of both conditions. The over-additive deficit in comparing small distances is associated with 2nd and 3rd grade children’s ability to compare two quantities in different modalities (e.g., dot sets and Arabic numerals). Based on the results, we cannot draw firm conclusions about the hypotheses regarding the causes of DD (see Table 1). We found that the higher the IQ, the lower the RT. IQ should be further investigated in future studies. It cannot be excluded that IQ plays a central role in solving this task. Nevertheless, the results extend the current state of research because mixed comparisons were rarely used, and when they were, the grade level of the DD and TD children was not systematically investigated.

### 4.4 Dot magnitude comparison (Panamath)

In line with previous findings (e.g., Rousselle and Noël, 2007; Landerl and Kölle, 2009; Decarli et al., 2023), children with DD showed no significant problems with non-symbolic magnitude comparisons, contradicting the ANS deficit hypothesis.

The causes of heterogeneous evidence regarding non-symbolic comparisons continue to be debated. Some authors discussed whether difficulties in non-symbolic comparisons are associated with severe mathematical difficulties (Wong et al., 2017). The discussion stemmed from the fact that studies found no impairments (e.g., Rousselle and Noël, 2007) using less stringent cutoff criteria to classify DD (*PR* = 15). In contrast, studies that have demonstrated differences (e.g., Piazza et al., 2010) used more stringent criteria (2 standard deviations below average, similar to the current study). We found no evidence of a deficit in DD despite the strict criterion, contradicting the assumption. Additionally, some authors argued that deficits in the non-symbolic comparison tasks result from poor mathematical development (see de Smedt et al., 2013), supporting the AD hypothesis. We investigated whether children’s grade level affects differences between children with and without DD. The present study could not confirm this assumption (similar to Skagerlund and Träff, 2014). However, Skagerlund and Träff (2014) investigated not only correctly answered trials (as the current study did), arguing that Weber fractions would be a more sensitive measure of performance on this task. Their findings showed that in line with the ANS hypothesis, DD children in 4th grade showed noisier ANS representations than TD children in 4th and 2nd grade. In other words, different test scoring methods also have an impact on results. Furthermore, methodological aspects of the task itself were discussed. Studies that used small ratios (e.g., Wong et al., 2017) found differences between children with and without DD compared to studies that used rather large ratios (e.g., Iuculano et al., 2008). We used small and large ratios and showed that DD and TD children did not differ in small ratios. However, the results showed that it is more challenging to compare small distances. In the TD group, it is clear that the children performed better at ratio 2 than at ratio 1. This distance effect is observed in all grades. In the DD group, this effect is less pronounced. DD children only performed better in 2nd grade. The developmental trajectories seem to differ, at least for ratios 1 and 2.

### 4.5 Dot magnitude (Panamath) vs. number comparison

To test whether DD children were disproportionately more impaired in symbolic comparisons, we contrasted children’s performance in symbolic vs. non-symbolic comparisons and distance effects for both tasks within one analysis. This methodical approach has rarely been used, and when it has been used, it has yet to be focused on children with DD (e.g., Holloway and Ansari, 2008). Similar to Kuhn et al. (2013), Landerl (2013), and Landerl and Kölle (2009), we found no interaction between group and distances. The result argues against a qualitative distance effect for children with DD. Similar to Schwenk et al. (2017), DD children required substantially longer RTs than TD children, particularly on symbolic comparisons. At first glance, this finding suggests a disproportionate impairment in symbolic tasks, consistent with the AD hypothesis. Pairwise comparisons of the interaction among task, group, and grade showed that TD and DD children did not differ at any grade level, suggesting that the disproportionate impairment could not be seen at grade level due to small group sizes. However, for both groups, we found that in the 2nd grade, symbolic magnitude comparisons resulted in significantly longer RTs than non-symbolic tasks. Thus, we reran the analysis without the 2nd graders and showed that the interaction between task and group effect disappeared. Against this background, the AD hypothesis seems to be tenable only in the 2nd grade.

### 4.6 Number sets

Consistent with other studies (e.g., Kuhn et al., 2013; von Wirth et al., 2021), DD children displayed difficulties in the number set task. DD children’s impairments were stable in different grades. There was no evidence suggesting a different developmental trajectory for children with DD. Consistent with previous research (e.g., Brankaer et al., 2014), children’s mapping skills developed across grade levels. Whether the deficits are more likely to result from deficient ANS/OTS or are indicative of AD remains unanswered, as both hypotheses predict deficits in this task.

### 4.7 Number line estimation

The results of this study are in line with previous research (e.g., Booth and Siegler, 2006; Decarli et al., 2023) and showed that children with DD displayed problems locating numbers on the number line. Children with DD were less accurate than TD children in 2nd grade; by the 3rd and 4th grades, there were no longer substantial group differences, suggesting DD children catch up and are merely developmentally delayed. Whether DD children in 3rd grade really catch up or if the task is not sensitive enough due to the small number range (0–100) remains unanswered, as Landerl (2013) examined number line estimation with a range of 0–1,000 and found that DD children became more accurate by 4th grade. In general, our results are in line with the defective ANS and AD hypotheses (see Wilson and Dehaene, 2007). In our study, children were only tested on locating written Arabic numerals on the number line. However, future studies should expand the task to rule out an ANS deficit, as Lafay et al. (2017) showed that DD children had no difficulty placing non-symbolic numerosities on the number line, suggesting that the mental number line or ANS is not damaged per se.

### 4.8 DD in 4th grade vs. TD in 2nd grade

Finally, we investigated whether the basic numerical profile of DD children in 4th grade is qualitatively different from that of TD children in 2nd grade or whether there is a developmental delay in DD children. The results revealed that DD children in 4th grade performed better than TD children in 2nd grade and caught up with TD children. Adapted from the ability-level-match design (Bradley and Bryant, 1978), this finding suggests that children with DD are developmentally delayed by less than 2 school years. As we found no interaction between tasks and group, the results do not indicate a task-specific DD profile, nor that DD children are disproportionately impaired. The present results confirm and complement the findings of previous studies. Skagerlund and Träff (2014) found no differences in RTs of basic numerical skills between DD children in 4th grade compared to a math ability-matched control group of TD children in 2nd grade, suggesting that the abnormalities are due to developmental delay. However, Skagerlund and Träff (2014) additionally used the Weber fraction and found that DD children in 4th grade had noisier ANS representations than TD children in 4th and 2nd grades. Thus, it may be useful in future to include the Weber fraction, which may be more sensitive to performance measures. Future studies should investigate whether this finding is robust and thus replicable in other samples. To extend the analyses and to be able to make statements about whether this indication of developmental delay persists in the long term, longitudinal studies comparing the basic numerical profiles of children with and without DD in higher grades would be interesting (e.g., DD children in 6th grade compared to TD children in 4th grade).

## 5 Limitations and suggestions for future research

Our results are more consistent with the ANS than with the AD (deficit) hypotheses and point against a deficit of the OTS. However, recent evidence suggests that there is no core cognitive deficit in DD. Individuals with DD may have deficits in basic numerical processing and domain-general cognitive abilities, but neither is necessarily present (Mammarella et al., 2021). Mammarella et al. (2021) argue that it is more fruitful to locate children with mathematical difficulties in a multidimensional space that reflects the severity of their difficulties and their relative position with respect to various domain-specific and cross-domain influences on mathematical performance. Subsequent studies should take this approach as a starting point for their analyses rather than focusing solely on domain-specific deficits.

Reading ability should also be considered because although recent evidence suggests that impairments in basic numerical skills are clearly associated with DD but not with a reading disorder (Raddatz et al., 2017; Decarli et al., 2023), we found that reading fluency affected basic numerical skills (e.g., dot enumeration). The same was true for IQ (e.g., mixed comparison).

To improve our understanding of the impairments of children with DD, it may be methodologically helpful to focus on a Bayesian statistical approach and to compare different competing models and theories.

Furthermore, our study compared independent groups in a cross-sectional design. Future longitudinal studies and research using the ability-level-match design are needed to definitively answer the intriguing question of whether DD children’s basic numerical abilities are qualitatively different from TD or whether DD children are developmentally delayed.

## 6 Conclusion

Consistent with the ANS deficit rather than the AD hypothesis, DD children consistently showed deficits in basic numerical skills. We found no evidence of deficits in subitizing, contradicting a disrupted OTS. The disproportionate impairment in processing numerosities from symbols and the qualitatively different distance effect in the mixed comparison task were moderated by grade level. Both grade level effects indicate that children with DD have a developmentally delayed rather than qualitatively different numerical processing. We found significant improvements in children’s performance with increasing grade levels on all tasks except Panamath. The results suggest developmental leaps between 2nd and 3rd graders. For mixed comparison, dot magnitude vs. number comparison, and number line, children with DD had a different developmental trajectory than TD children. However, the results indicate that children with DD have a developmentally delayed rather than a qualitatively different basic numerical profile. The only disproportionate DD impairment that was not moderated by grade level relates to the counting range of the dot enumeration task. This result emphasizes the potential of (pre)-school identification, prevention, and intervention initiatives. Verbal counting indicates risks related to math abilities and predicts math achievement in the long term (Koponen et al., 2019). This learned skill (see Krajewski and Schneider, 2009) builds on innate core systems (von Aster and Shalev, 2007). Therefore, interventions should start before further basic numerical skills develop poorly due to defective innate core systems. Moraske et al. (2019) showed that early intervention of basic numerical skills in kindergarten leads to improved later math performance in DD at-risk children and reduces the likelihood of the onset of dyscalculia.

Most of the evidence points to a developmentally delayed basic numerical profile in DD. If teachers have evidence-based knowledge about the causes (von Aster and Shalev, 2007) and deficits (e.g., Butterworth, 2010; Kuhn et al., 2013) in DD, they could locate the deficits of the DD child in the developmental stage (e.g., Krajewski and Schneider, 2009) and initiate adaptive interventions according to the response-to-intervention approach (Voß et al., 2014). This approach has great potential, as appropriate interventions can improve children’s performance significantly (Chodura et al., 2015). However, the first step to appropriate intervention is to identify children with DD who are at risk. The fact that even basic numerical skill tasks discriminate between children with and without DD in primary school underlines the importance of teachers including basic numerical skills when identifying or supporting children with DD. To support teachers in identifying children with DD, future studies should focus on the development of simple screening tools.

## Data availability statement

The datasets presented in this article are not readily available because the data will be used for further analyses and publications. Requests to access the datasets should be directed to tobias.kuhn@tu-dortmund.de.

## Ethics statement

The studies involving involving human participants were reviewed and approved by the Ethikkommission des Fachbereichs 7, Psychologie und Sportwissenschaft, Westfälische Wilhelms-Universität Münster (ethics committee of Faculty 7 - Psychology and Sports Sciences, University of Münster). The studies were conducted in accordance with the local legislation and institutional requirements. Written informed consent for participation in this study was provided by the participants’ legal guardians/next of kin.

## Author contributions

J-TK: contributed the idea for writing this manuscript, funding acquisition, and data collection. J-TK and SL: statistical analysis. SL: created the basic structure of the manuscript (lead). J-TK and FK: provided the writing oversight and revision of the draft. All authors contributed to the article and approved the submitted version.
