# Erratum: Delayed development of basic numerical skills in children with developmental dyscalculia

**DOI:** 10.3389/fpsyg.2024.1384448

**Published:** 2024-02-20

**Authors:** Frontiers Production Office

**Affiliations:** Frontiers Media SA, Lausanne, Switzerland

**Keywords:** developmental dyscalculia, basic numerical skills, domain-specific deficits, dot enumeration, magnitude comparison, number sets, number line

Due to a production error, several corrections requested by the author during proofing were not implemented in the final published article.

In section *2.2.2 Intelligence*, amendments were made to correct the types of dash and bracket used here. The corrected paragraph is shown below.

The non-verbal intelligence of 2nd and 3rd graders was examined by three subtests of the *Grundintelligenztest Skala 1-Revision* (CFT 1-R; Engl.: Culture Fair Intelligence Test 1-R Scale 1; Weiß and Osterland, 2013): series completion, classification, and matrices (*r*_tt_ = 0.95). The 4th graders were assessed using four subtests of the *Grundintelligenztest Skala 2-Revision* (CFT 20-R; Engl.: Culture Fair Intelligence Test 20-R Scale 2; Weiß, 2006): series completion, classification, matrices, and topologies (*r*_tt_ = 0.80). The test score (IQ) was calculated from the subscales used in each case.

Additionally, there was an error in section *2.2.3 Reading fluency*. Instead of “The children were presented with a list of 70 simple sentences containing correct and incorrect statements (e.g., “you can drink tea” or “cherries can talk”)” the sentence should be: “The children were presented with a list of 70 simple sentences containing correct and incorrect statements (e.g., “bananas can talk”).” The corrected paragraph is shown below.

Reading fluency was measured using the paper-based Salzburger Lese-Screening für die Klassenstufen 1–4 (SLS 1–4; Engl.: Salzburg reading screening test for grades 1–4; parallel-forms reliability = 0.90; Mayringer and Wimmer, 2003). The children were presented with a list of 70 simple sentences containing correct and incorrect statements (e.g., “bananas can talk”). Within 3 min, as many sentences as possible had to be read and judged with regard to their correctness. The test score depends on the total number of sentences judged correctly.

Furthermore, the title of Table 9 should be written as “TABLE 9 Results of the linear mixed model using group, grade, reading fluency, and intelligence to predict children's performance on number line,” and the sentence “In contrast, studies that have demonstrated differences (e.g., Piazza et al., 2010) used more stringent criteria (2 *SD* below average, similar to the current study).” in section *4.4 Dot magnitude comparison (Panamath)*, should be: “In contrast, studies that have demonstrated differences (e.g., Piazza et al., 2010) used more stringent criteria (2 standard deviations below average, similar to the current study).”

The Supplementary Material file has also been republished in PDF format.

The publisher apologizes for these mistakes.

The original article has been updated.

## References

[B1] Mayringer H. Wimmer H. (2003). Salzburger Lese-Screening für die Klassenstufen 1–4 (SLS 1–4) [Salzburg Reading Screening Test for Grades 1–4]. Bern: Huber.

[B2] Piazza M. Facoetti A. Trussardi A. N. Berteletti I. Conte S. Lucangeli D.. (2010). Developmental trajectory of number acuity reveals a severe impairment in developmental dyscalculia. Cognition 116, 33–41. 10.1016/j.cognition.2010.03.012 20381023

[B3] Weiß R. H. (2006). Grundintelligenztest Skala 2 - Revision (CFT 20-R) mit Wortschatztest und Zahlenfolgentest - Revision (WS/ZFR). [Culture Fair Intelligence Test 20-R Scale 2 with vocabulary and numerical order test]. Göttingen: Hogrefe.

[B4] Weiß R. H. Osterland J. (2013). Grundintelligenztest Skala 1 – Revision (CFT 1-R). [Culture Fair Intelligence Test, Scale 1-R Scale 1]. Göttingen: Hogrefe.

