# Supplementary material for: Delayed development of basic numerical skills in children with developmental dyscalculia
Source: Front Psychol. 2024 Jan 11;14:1187785. doi: 10.3389/fpsyg.2023.1187785 (PMC10810128; doi:10.3389/fpsyg.2023.1187785)
Supplement: Supplementary file 1 [file Table_1.pdf]

SUPPLEMENTARY MATERIAL

Table 12

Descriptive results, scores on basic numerical tasks by grade and mathematical ability

	2nd grade		3rd grade		4th grade	
Details	TD	DD	TD	DD	TD	DD
DE sub. (1–3)	101.94 (12.70)	115.75 (18.36)	93.73 (11.53)	98.17 (9.56)	89.51 (9.51)	90.07 (6.03)
DE cont. (4–9)	101.50 (11.36)	114.18 (19.87)	93.52 (11.27)	100.63 (11.10)	89.03 (10.33)	98.21 (11.17)
DE sub. (1–4)	110.01 (15.20)	136.38 (25.02)	97.25 (11.68)	106.69 (12.23)	89.41 (8.88)	103.25 (11.56)
DE cont. (5–9)	107.26 (14.99)	133.97 (14.77)	97.38 (13.69)	114.05 (15.67)	90.76 (10.94)	105.27 (19.66)
NC small distances	105.01 (16.58)	120.90 (15.75)	95.25 (10.50)	105.49 (12.69)	88.63 (8.41)	103.01 (12.04)
NC large distances	105.26 (15.73)	125.15 (21.30)	95.15 (10.21)	102.81 (11.41)	88.69 (8.43)	102.46 (13.42)
MC small distances	104.34 (14.69)	117.03 (20.55)	96.24 (12.69)	104.84 (17.01)	92.73 (11.70)	91.51 (9.68)
MC large distances	103.87 (14.91)	120.75 (31.07)	96.25 (10.07)	103.88 (17.24)	91.33 (10.20)	98.87 (12.36)
Panamath, ratio 1.2	102.60 (15.88)	104.89 (15.87)	97.7 (11.16)	104.33 (29.27)	94.68 (7.92)	106.47 (22.61)
Panamath, ratio 1.4	102.25 (15.01)	105.13 (13.60)	97.95 (11.32)	106.63 (29.90)	94.54 (8.41)	104.82 (26.89)
Panamath, ratio 1.6	101.98 (15.13)	105.05 (15.90)	98.64 (12.41)	105.85 (27.16)	93.53 (9.76)	105.1 (22.04)
Panamath, ratio 2.6	101.77 (14.50)	102.8 (9.81)	98.47 (11.66)	108.67 (36.05)	95.13 (9.09)	101.58 (19.06)
NS	96.24 (13.20)	80.33 (11.18)	104.09 (14.03)	91.17 (12.80)	111.74 (12.24)	95.21 (15.22)
NL	104.70 (16.36)	124.00 (18.74)	95.06 (11.07)	103.39 (12.73)	91.61 (6.35)	99.70 (14.26)

Note. Scaling for all measures (*M*: 100/*SD*: 15). DE sub = Dot enumeration subitizing range, DE cont. = Dot enumeration counting range, NC = Number comparison, MC = Mixed comparison, Panamath = the median of the children's RTs for correct answers, NS = Number sets, NL = Number line estimation.
